# Me31B: a key repressor in germline regulation and beyond

**DOI:** 10.1042/BSR20231769

**Published:** 2024-04-30

**Authors:** Ming Gao

**Affiliations:** Biology Department, Indiana University Northwest, Gary, IN, U.S.A.

**Keywords:** Germ granules, Germline, Me31B, Neuronal granules, Ribonucleoprotein (RNP), RNA helicase

## Abstract

**M**aternally **E**xpressed at **31B** (Me31B), an evolutionarily conserved ATP-dependent RNA helicase, plays an important role in the development of the germline across diverse animal species. Its cellular functionality has been posited as a translational repressor, participating in various RNA metabolism pathways to intricately regulate the spatiotemporal expression of RNAs. Despite its evident significance, the precise role and mechanistic underpinnings of Me31B remain insufficiently understood. This article endeavors to comprehensively review historic and recent research on Me31B, distill the major findings, discern generalizable patterns in Me31B’s functions across different research contexts, and provide insights into its fundamental role and mechanism of action. The primary focus of this article centers on elucidating the role of *Drosophila* Me31B within the germline, while concurrently delving into pertinent research on its orthologs within other species and cellular systems.

## Drosophila germline and maternal effect gene *me31B*

**Figure 1:**
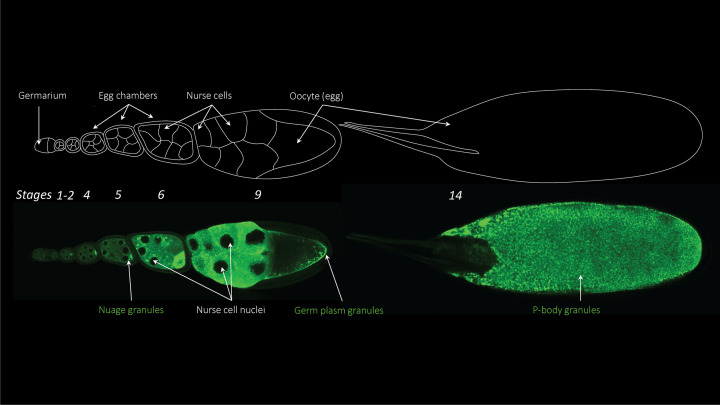
*Drosophila* oogenesis and Me31B expression in the egg chambers Me31B-GFP expression is visualized in an ovariole expressing GFP-tagged wild-type Me31B [19]. The top image is a sketch outlining the egg chambers and nurse cells while the bottom image shows Me31B-GFP observed using confocal microscopy.

*Drosophila melanogaster* germline is a useful model for exploring the genetic factors involved in animal germline development [1–7]. Within the female germline, the ovaries consist of a series of tubular structures named ovarioles, each of which houses a chain of developing egg chambers at various stages of oogenesis (which range from 1 to 14, Figure 1). This process begins with the division of germline stem cells in the germarium at the tip of each ovariole, resulting in interconnected cyst cells. Each cyst includes 15 nurse cells and one oocyte, surrounded by a layer of somatic follicle cells, forming an egg chamber. Within each egg chamber, the nurse cells synthesize nutrient molecules – including maternally expressed RNAs and proteins – and deposit them into the developing egg, supporting growth and maturation. These maternal RNAs and proteins form a complex gene-expression-regulation program, guiding the germline through oogenesis, embryogenesis, and eventual development into the next generation [4,8–14]. The protein **M**aternally **E**xpressed at **31B** (Me31B) is a crucial component among the maternal gene products for the development of germline [15]. Its orthologs in other animals also appear to possess this same significance [16–18].

## Historic background of *me31B* gene

The discovery of the second maternally expressed *Drosophila* gene, *me31B*, was made by De Valoir et al. [20]. Similar to the first maternal gene *vas* (encodes another RNA helicase Vas), *me31B* transcripts are predominantly found in *Drosophila* female germline cells: nurse cells, oocytes, and early embryos. As a result, *me31B* was named after this maternal expression pattern and its genomic location at the 31B region of the second chromosome [20]. In the early study, *me31B* expression was only detected in the female germline due to the limited detection sensitivity. However, recent studies have discovered *me31B*'s expression and roles in the male germline and various somatic tissues.

## The Me31B helicase family and Me31B’s domain/motifs

Me31B belongs to the DEAD-box RNA helicase family, a large group of proteins that use the energy from ATP hydrolysis to unwind RNAs during various RNA metabolism processes [21–28]. This is indicated by the presence of the signature Asp-Glu-Ala-Asp (DEAD) sequence and other structural features [20]. Me31B and its orthologs are classified under the evolutionarily conserved subfamily of Me31B/DDX6-like, ATP-dependent, DEAD-box, RNA helicases. This subfamily includes various members, including human DDX6/RCK, mouse p54, frog Xp54, worm CGH-1, yeast Dhh1p, and others, with a sequence similarity of 60–80% between each other [15,18,29]. For simplicity, we commonly use the term Me31B as a generic reference to the aforementioned proteins, unless there is a need to specify.

The protein Me31B contains several conserved domains, motifs, and amino acids important for its functions. Table 1 summarizes these functional domains and motifs’ locations, known/hypothetical functions, and level of conservation. Me31B domains and motifs have been categorized into six groups based on functions and sequence characteristics. Group One consists of two large RecA-like domains (N-terminal RecA-like and C-terminal RecA-like domains) that potentially cooperate to unwind RNAs, along with four motifs (DVLARAK, DEAD-box, SAT, and HRIGR motifs) that are critical for ATPase/helicase activities. Group Two contains 11 highly conserved motifs (Q, I, Ia, GG, Ib, II, III, IV, QxxR, V, and VI motifs) with poorly understood functions. Group Three contains four amino acids (H333, R334, C351, and S352) that are crucial for human development as deduced from human ortholog DDX6. Group Four has two intrinsically disordered regions (IDRs) that could affect Me31B protein self-aggregation. Group Five includes two hypothetical nuclear localization and export signal sequences (NLS and NES, both deduced from homologous sequences of DDX6). Group Six contains 16 additional motifs that facilitate Me31B’s interaction with other proteins. The above domains and motifs are believed to play important roles in Me31B functions for regulating germline RNA stability, localization, and translation, which, in turn, contributes to proper germline development.

**Table 1:** *Drosophila* Me31B domains and motifs

Me31B domains and motifs*	Extent of conservation**	Known or hypothetical functions	Reference
***Group One: Large functional domains and motifs participating in ATPase and RNA helicase activity***			
**N-terminal domain** (1–267)	Conserved RecA-like domain in humans and other species	N-terminal RecA-like domain; ATP binding; Helicase activity; P-body assembly; Female fertility	[18,30,19]
**C-terminal domain** (268–459)	Conserved RecA-like domain in humans and other species	C-terminal RecA-like domain; RNA translational repression; Protein binding; P-body assembly; Female fertility	[19, 30, 31]
**DEAD box** (207–210, within II motif)	100% in humans and other species	ATP-binding, RNA binding, RNA translational control, protein binding; Female fertility	[19, 29, 30,32]
**SAT** (238–240, same as III motif)	100% in humans and other species	Helicase activity	[18,30]
**HRIGR** (381–385, within VI motif)	100% in humans and other species	ATP binding, RNA binding, helicase activity, RNA translational repression, p-body accumulation and P-body assembly; Female fertility	[19, 29, 30]
**DVLARAK** (97–103, partial overlap with I motif)	93% of human and other species	RNA binding, translational control, P-body assembly; Female fertility	[30,19]
***Group Two: Motifs highly conserved in the Me31B/DDX6 family in humans and other species with mostly unknown functions***			
**Q** (77–85)	94% in humans and 89–94% in other species	Unknown	[18]
**I** (102–109)	94% in humans and 94–100% in other species	Unknown	[18]
**I_a_** (134–141)	100% in humans and other species	Unknown	[18]
**GG** (162–163)	100% in humans and other species	Unknown	[33]
**I_b_** (180–186)	94% in humans and 94–100% other species	Unknown	[18]
**II** (204–210, contains DEAD box)	94% in humans and 94–100% in other species	Containing the DEAD box	[18]
**III** (238–240, same as SAT motif)	100% in humans and other species	Helicase activity	[18,30]
**IV** (299–301)	100% in humans and other species	Unknown	[18]
**QxxR** (331–334)	100% in humans and other species (for Q and R)	Unknown	[16]
**V** (356–360)	100% in humans and other species	Unknown	[18]
**VI** (380–390, contains HRIGR motif)	100% in humans and 95–100% in other species	Unknown	[18]
***Group Three: Motifs conserved in humans and their mutations result in human developmental defects***			
**H333** (within QxxR motif)	100% in humans and other species	The mutation causes cognitive and developmental delays and defects (in DDX6)	[16]
**R334** (within QxxR motif)	100% in humans and other species	The mutation causes cognitive and developmental delays and defects (in DDX6)	[16]
**C351**	100% in humans and other species	Mutation causes cognitive and developmental delays and defects (in DDX6)	[16]
**S352**	50% in humans and 50–100% in other species	Mutation causes cognitive and developmental delays and defects (in DDX6)	[16]
***Group Four: Intrinsically disordered regions (IDR)***			
**N-terminal IDR** (1–53)	Presence, length, and sequence vary among species	Protein aggregation regulation	[34]
**C-terminal IDR** (431–459)			
***Group Five: Hypothetical signal peptides involved in nuclear import/export***			
**Nuclear localization signal** (NLS, 35–48, hypothetical)	Presence, length, and sequence vary among species	Nuclear localization signal (in DDX6)	[35]
**Nuclear export signal** (NES, 113–122, hypothetical)		Nuclear export signal (in DDX6)	
***Group Six: Other protein- and RNA-binding motifs***			
**FDF motif-binding** (285–289, part of EDC3 and Tral binding region motif and other motifs)	100% in humans and other species	FDF-domain binding motif; Aggregation status	[19,36]
**W pocket** (310–314)	100% in humans and other species	4E-T, EDC3, and LSM14A binding site (in DDX6)	[36]
**Protein binding patch 1** (281–294, partially overlap with EDC3 and Tral binding region motif and other motifs)	100% in humans and 94–100% in other species	Potential FDF-binding site for binding Pat1 and EDC3 (in DDX6)	[35,37]
**Protein binding patch 2** (405–412, partially overlaps with Y401-L407 motif)	63% in humans and 50–63% in other species	Interaction with EDC3 (in DDX6)	[33,35]
**Protein binding patch 3** (304–307)	75% in humans and 50–75% in other species	4E-T and PATL1 binding site (in DDX6)	[35]
**NOT1 binding specificity motif** (290–296, partially overlaps with EDC3 and Tral binding region motif and other motifs)	93% in humans and 86–93% in other species	Provides binding specificity to NOT1; RNA translational repression (in DDX6)	[38]
**EDC3 and Tral binding motif** (274–292, partially overlaps with Protein binding patch 1 and other motifs)	95% in humans and 92–95% in other species	EDC3/Tral binding; Localization to P-body; RNA translational repression	[30,33]
**F63** **–** **L70**	87% in humans and 81–87% in other species	Interaction with eIF4E-3 isoform	[39]
**R347**	100% in humans and other species	Needed for binding to CNOT1 (in DDX6)	[35,37,38]
**R80**	50% in humans and 0–50% in other species	Potential symmetrically demethylated arginine (SDMA) interacting with Tud-domain proteins	[40]
**R156**	50% in humans and other species	Potential SDMA interacting with Tud-domain proteins	[40]
**R254**	0% in humans and 0–100% in other species	Potential SDMA interacting with Tud-domain proteins	[40]
**RG (R357 and G358)**	100% in humans and other species	Involved in de-capping (in Dhh1p)	[41]
**K108**	100% in humans and other species	ATP-binding and RNA translational control (in Bel helicase)	[42]
**Y273**	100% in humans and other species	Potential Phosphorylation site; function unknown (in DDX6)	[35]
**Y401-L407** (partially overlaps with Protein binding patch 2 motif)	79% in humans and 71–79% in other species	Interaction with eIF4E-1	[39]

* Note that certain domains and motifs’ sequences and functions are deductions from Me31B orthologs in other species.

** Extent of conservation is calculated with six species: *C. elegans*, planarian, *Drosophila*, *Xenopus*, mouse, and human. We counted a mismatch between two chemically different amino acids like arginine (R) and Valine (V) as a mismatch, while a mismatch between two chemically similar amino acids like arginine (R) and lysine (K) was counted as half a match.

## Analysis of Me31B’s roles in the germline

To investigate the role of Me31B in the germline, a range of different *me31B* alleles have been generated using traditional and targeted-motif-mutation approaches.

### Loss-of-function alleles

Nakamura et al. initially produced three loss-of-function alleles of *me31B*, specifically, *me31B*^Δ^*^1^*, *me31B*^Δ^*^2^*, and *me31B*^Δ^*^3^*, which resulted from partial deletions of the *me31B* gene [15]. The three alleles all caused recessive lethality, with homozygous mutants failing to develop during the second or third-larval stages, despite no visible morphological defects. These findings demonstrate that *me31B* is an essential gene for *Drosophila*. To gain a deeper understanding of its roles in the germline, the researchers used the m*e31B*^Δ^ allele to create germline clone strains, where animals carried homozygous *me31B*^Δ^ mutation in the germline but otherwise wild type. In the germline clone ovaries, egg chambers rarely developed beyond Stage 10, with various developmental defects such as egg chamber degeneration and germline cell membrane collapse. Strong *me31B* germline knockdown strains showed similar oogenesis defects [40], further emphasizing the importance of *me31B* in oogenesis. Crucially, the loss of *me31B* in the germline led to the abnormal translational activation of key germline-development RNAs, *osk*, and *BicD*, suggesting that Me31B acts as a translation repressor.

### Loss of one *me31B* gene copy

Although the loss of one *me31B* gene copy (m*e31B*^Δ^ heterozygotes) does not seem to affect growth in *Drosophila*, the presence of heterozygosity for both m*e31B*^Δ^ and other crucial germline or neuron development genes could result in observable phenotypes in the corresponding tissues. In the germline, a reduction in germ cell number phenotype was observed in early embryos when one *me31B* gene copy was lost. Additionally, when one *me31B* gene copy and one copy of germ-plasm-protein genes like *vas* or *tud* were lost simultaneously, a more severe reduction was observed [43]. This implies that *me31B* has a genetic interaction with germ plasm genes, and the interaction is crucial for proper germ cell formation. The effects of *me31B*^Δ^ heterozygosity in neurons will be discussed further below (see Me31B in The Soma).

### Targeted-domain mutations of Me31B

Target-motif-mutations of *me31B* were generated to investigate the functions of specific Me31B motifs. To investigate the role of Me31B helicase activity, three helicase-activity-mutation alleles (*me31B^E208A^*, *me31B^DVLAAAA^*, and *me31B^R385Q^*) were generated using CRISPR through point mutations in those motifs: D**E**AD→D**A**AD, DVLA**R**A**K**→DVLA**A**A**A**, and HRIG**R**→HRIG**Q** [19]. These three alleles cause female sterility dominantly. The sterility is associated with various oogenesis and embryogenesis defects such as egg chamber degenerations, dorsal appendage abnormalities, and unhatchable eggs. This underscores the crucial role of Me31B’s helicase activity in germline development and fertility. At the molecular level, the dominant helicase-activity mutations reduce Me31B protein levels and alter the germ plasm localization pattern of germline mRNA *nos* (encoding protein Nos, a key and conserved regulator involved in embryo patterning and germ cell development [1,44–46]). This suggests that Me31B’s helicase activity is involved in stabilizing the protein and modulating substrate RNAs.

To investigate the functions of Me31B’s N-ter domain, C-ter domain, and FDF-binding motif (a partner protein binding motif), three additional target-motif-mutation alleles have been created. These are *me31B^N-ter^* (containing N-terminal AA 1–276), *me31B^C-ter^* (containing C-terminal AA 277–459), and *me31B^FDF^* (point mutations in the FDF-binding motif which mediates Me31B’s interaction with partner proteins like Tral and Edc3) [19]. Homozygous *Drosophila* carrying the three alleles (*me31B^N-ter^*, *me31B^C-ter^*, and *me31B^FDF^*) are viable and can complete oogenesis, indicating that these are weak alleles of *me31B*. However, homozygotes of the three alleles display varying degrees of fertility reduction, with *me31B^C-ter^* causing complete sterility in a recessive manner (associated with embryo patterning defects). At the molecular level, the *me31B^N-ter^* and *me31B^C-ter^* mutations reduce Me31B protein levels to varying degrees, while all three mutations (*me31B^N-ter^*, *me31B^C-ter^*, and *me31B^FDF^*) alter Me31B intracellular localizations in different ways. This suggests that Me31B’s N-ter domain, C-ter domain, and FDF-binding motif all contribute distinct functions to Me31B stability, subcellular localization, and ultimately, fertility.

## Me31B expression and Me31B RNP granules

*me31B* transcripts are present throughout all stages of oogenesis from early to late egg development. The transcripts are found in all types of germline cells, including nurse cells (with a burst of *me31B* transcription at early-to-mid oogenesis, around stage 6-7), developing oocytes, and early embryos (0–4 h). The transcripts exhibit a uniform, cytoplasmic distribution in these cells. However, the transcripts are no longer detectable in later embryos (after 4 h) [20].

The expression level of Me31B protein parallels that of the *me31B* mRNA. Me31B is detected in early oogenesis in the germarium (the part of the ovary that contains undifferentiated germline stem cells) and remains at a relatively low level during the early stages of oogenesis (stages 1–5). However, there is a significant increase in Me31B around stages 6–7, which is consistent with elevated transcription. Me31B remains at a high level throughout the rest of oogenesis and declines to non-detectable levels in early embryogenesis [15]. Unlike mRNA, Me31B proteins have specific subcellular localization and aggregation patterns [15,40,47] (Figure 1, bottom). They are observed in the cytoplasm, not the nuclei, of both nurse cells and developing oocytes. Me31B is concentrated in oocytes, possibly transported from nurse cells via ring canals (channel-like structures that connect the cytoplasm between the nurse cells and oocytes). In nurse cells and oocytes, Me31B often takes the form of aggregated granules. These granules accumulate in specific regions, such as the peri-nuclear regions of nurse cells (nuage), the cortex of mid-stage oocytes, and the germ plasm area at the posterior pole of mid-to-late-stage oocytes (germ plasm is the special cytoplasm that contains germ-cell-formation determinants). The granules disperse and become uniformly distributed in late-stage eggs and early embryos before disappearing by the cellular blastoderm stage of embryos.

The Me31B-containing granules are germline ribonucleoproteins (RNPs), which are membraneless and liquid-like condensates composed of germline RNAs and proteins [1,4,11,12,48–51]. These granules are classified into three major groups based on their molecular compositions, subcellular locations, and morphologies: nuage granules found around nurse cell nuclei, P-body/sponge body granules dispersed in the cytoplasm of nurse cells, developing oocytes, and early embryos, and germ plasm granules at the posterior pole of developing oocytes/embryos. Me31B plays an important role in regulating the fate of RNAs in the RNPs by interacting with the RNA and protein constituents, which is an essential process for germline development [9,34,47,52–54].

## Functions of Me31B in RNP granules

### Physical properties and dynamics of the granules

RNP granules containing Me31B, such as P-bodies, sponge bodies, nuage granules, and germ plasm granules, display physical properties similar to sticky, liquid droplets and are referred to as biomolecular condensates [1,11,34,48,55–57]. A recent study used Me31B-labeled P-bodies during late oogenesis to early embryogenesis to illustrate the general physical properties of germline RNPs and how the properties change with different cellular contexts [34].

In mature oocytes, P-bodies labeled with Me31B are widespread and universally distributed in the bulk oocyte cytoplasm. These micro-sized structures vary in morphology and are highly dynamic, constantly rearranging through fusion and fission. The P-body structure is maintained by a variety of physicochemical and biological forces, including hydrophobic interactions, salt concentrations, RNAs, the cytoskeleton, and protein–protein interactions (particularly the protein-protein interactions between Me31B and Tral). Moreover, recombinant Me31B proteins can form spherical condensates in the presence of a crowding agent, and Me31B’s Intrinsically Disordered Regions (IDRs) play a role in modulating the self-aggregation process, suggesting that the IDRs may regulate Me31B aggregation within the P-bodies.

As the developmental stage progresses from mature oocyte to early embryo, the physical properties of Me31B-labeled P-bodies change. P-bodies in mature oocytes maintain typical morphologies and undergo slow rearrangement, with very limited exchange between Me31B in the P-bodies and the cytoplasm. This limited exchange suggests that the P-bodies function as storage units for oocyte RNAs. However, in early embryos after egg activation, the P-bodies become much more dynamic. They disperse into smaller condensates, contain more highly mobile Me31B, and dissociate from stored maternal RNAs like *bicoid (bcd)*. The change from the arrested state in mature oocytes to the dynamic state in early embryos is believed to occur upon egg activation, facilitating the disintegration of the P-bodies and the release of the stored RNAs for translation, enabling further embryogenesis.

### Me31B interactome in the RNPs

**Figure 2:**
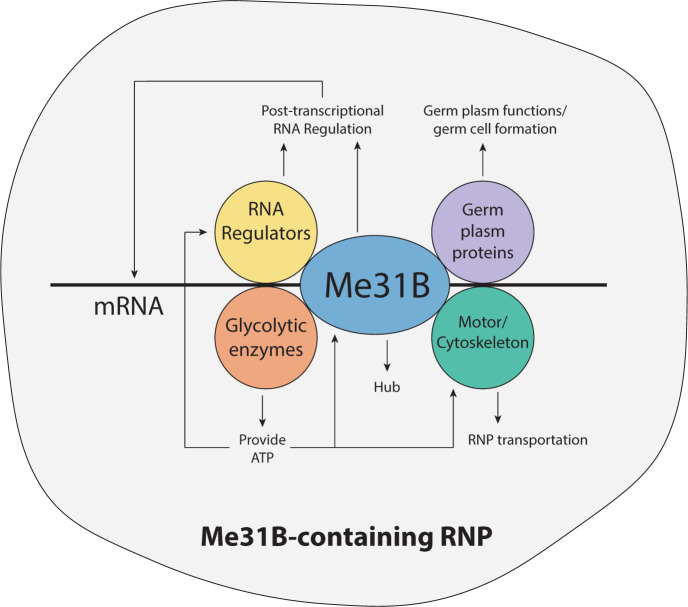
The model of a Me31B complex in germline RNP In this model of Me31B-containing RNP, the Me31B protein assumes a pivotal role as the central hub, orchestrating interactions with four groups of protein partners: RNA regulators, cytoskeleton/motor proteins, glycolytic enzymes, and core germ plasm proteins. Among these, the RNA regulators, including Me31B itself, provide post-transcriptional RNA regulations. Cytoskeleton/motor proteins facilitate the transportation of the RNP. Glycolytic enzymes contribute ATP resources to power the activities of other proteins within the assembly. Lastly, core germ plasm proteins play integral roles in the assembly of germ plasm/nuage and the formation of germ cells. The figure is adapted from DeHaan et al. [40].

To gain insight into the functions of Me31B complexes in RNP granules, a proteomics approach was implemented to isolate Me31B complexes from *Drosophila* ovaries. Through this approach, the Me31B protein interactome was comprehensively identified [40], revealing the presence of proteins from four functional groups: RNA regulators (repressors like Cup and Tral; degraders like PCM and EDC3), cytoskeleton and motor proteins (like dynein, kinesin, and tubulin components), glycolytic enzymes, and core germ plasm proteins involved in germ plasm assembly and germ cell formation (like Vas, Aub, and Tud). This led to the development of a model for a Me31B-centered functional unit in the RNP granules, with Me31B serving as the central hub (Figure 2). The proposed model outlines the various components that work in conjunction with Me31B, highlighting their individual purposes and relationships. First, Me31B collaborates with RNA repressor partners to enable post-transcriptional regulations over the RNAs. Second, cytoskeleton/motor proteins act as moving tracks and energy sources for the transportation of the granules. Third, glycolytic enzymes provide ATP to support the functioning of RNA helicases and motor proteins. Fourth, Me31B interacts with core germ plasm proteins, which hints at its possible roles in the assembly and function of nuage and germ plasm granules.

In a subsequent investigation utilizing a comparable proteomics approach, a Me31B early-embryo interactome was acquired, which corroborated the suggested model above by revealing the same four protein groups. In addition, when compared with its ovary interactome counterpart, the Me31B early-embryo interactome showed a significant reduction of core germ plasm proteins, supporting the notion that Me31B RNP granules differ in composition in different cellular contexts [47].

To understand Me31B’s RNA interactome, a study utilized an RNA immunoprecipitation + sequencing (RIP-seq) approach to comprehensively identify Me31B-bound mRNAs in *Drosophila* early embryos. The results revealed that transcripts from almost all expressed genes were enriched in the Me31B precipitates [58]. This suggests that Me31B may bind most if not all, expressed germline mRNAs without specificity. This interpretation was further supported by a Me31B-germline mRNA co-staining experiment, which found seven representative mRNAs (*osk*, *BicD*, *bcd, nos*, *orb*, *Pgc*, and *gcl*) to colocalize with Me31B granules in various stages of egg chambers [15]. Additionally, the RIP-Seq study has revealed that Me31B has different effects on its bound mRNAs depending on the cellular contexts [58]. To elaborate, during the early stages of embryonic development (0-1 hour, before the Maternal to Zygotic Transition (MZT), an event that marks the shift in developmental control from maternal factors to zygotic gene expression), Me31B binds to nearly all transcripts and inhibit their translation. However, as the embryos go through MZT (1–5 h), the transcripts bound by Me31B undergo progressive changes, and the binding shifts from repressing translation to destabilizing the mRNAs. This highlights the dynamic RNA interactome and functions of Me31B throughout germline development.

### Me31B repressor complex on *nos* mRNA

**Figure 3:**
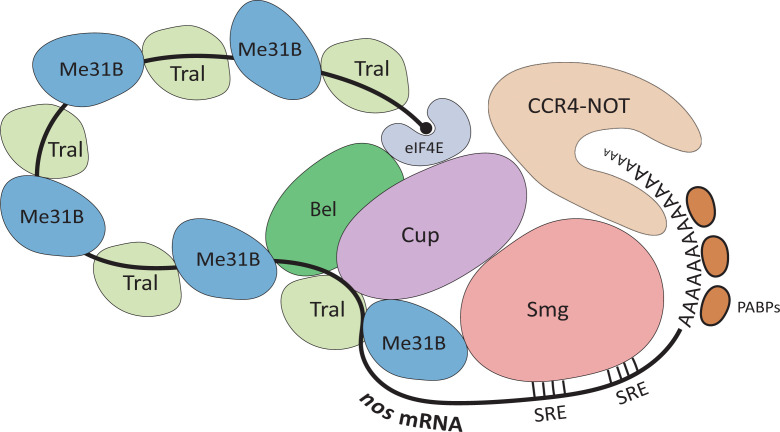
The model of a Me31B-containing repressor complex on *nos* mRNA The model involves Smaug (Smg), functioning as an RNA repressor/degrader, binding to Smaug Recognition Elements (SREs) in the 3′ UTR of *nos* during embryonic Maternal to Zygotic Transition (MTZ). Smg recruits Cup, which then interacts with eIF4E, Tral, and Me31B. Multiple Me31B-Tral complexes coat *nos* RNA, hindering translation. Additionally, Me31B or eIF4E may also engage Belle (Bel) to further suppress *nos* RNA. Finally, Me31B and/or Smaug recruit the CCR4-NOT deadenylation complex, leading to *nos* RNA degradation. The figure is adapted from Gotze et al. [54].

To explain how a Me31B-containing repressor complex can suppress and break down mRNAs during germline development, we reference the *nanos (nos)* mRNA repression complex model proposed by Gotze et al [54] and its associated sources. The intricate suppression and degradation of *nos* mRNA is crucial for the proper timing and location of Nos protein production, which ultimately contributes to *Drosophila* embryo body patterning and germ cell formation [10,59–61]. This repression mechanism relies on the collaboration of Me31B and other RNA repression and degradation factors, such as Smaug (Smg), Belle (Bel), Cup, Tral, the CCR4-NOT complex, and more (Figure 3) [31,42,54,62–65]. The model begins with Smaug (Smg), an RNA repressor/degrader during the MTZ of embryogenesis, binding to the two Smaug Recognition Elements (SREs) in the 3′ UTR of *nos*. Smg then recruits Cup, which further binds eIF4E, Tral, and Me31B directly or indirectly. Me31B interacts with Tral via its FDF-binding motif to Tral’s FDF motif, and the Me31B-Tral complex along the length of *nos* RNA, coating it and inhibiting its translation. Me31B or eIF4E may also bind Belle (Bel), another repressor helicase, to cooperatively repress *nos* RNA. Finally, Me31B and/or Smaug recruits the deadenylation complex CCR4-NOT to digest the *nos* from its 3′ poly-A tail and destabilize the RNA for degradation. It is important to note that different repression models may be at work on *nos* mRNA at different developmental stages. For example, in late oogenesis rather than early embryogenesis, *nos* repression relies on Glorund (Glo). It directly binds to *nos* mRNA at a 3′-UTR sequence different from the SREs and recruits another repressor dFMRP to inhibit *nos* translation [46,66–68]. Despite this, the example of *nos* repressor complex suggests that Me31B may play repressor roles on germline mRNAs by directly coating the mRNA to shield it from translational machinery and facilitating the communication/recruitment of other mRNA-specific (like Smg) or non-specific (like Cup and Tral) repressors.

### Interaction between Me31B-containing P-bodies and U bodies

Recent research revealed physical and genetic connections between Me31B-containing P-bodies and other RNP structures like U bodies. U bodies, named after their Uridine-rich small nuclear ribonucleoproteins (U snRNPs), are believed to be the cytoplasmic sites of snRNP biogenesis, assembly, and storage before they are transported into the nucleus for mRNA processing [69]. While distinct entities within *Drosophila* germline cells [69], P-bodies and U bodies exhibit evidence of mutual interaction. For instance, although not all P-bodies are linked with U bodies, U bodies consistently associate with P bodies [69,70]. Additionally, mutations affecting key components of both structures (such as SMN in U bodies and Cup in P-bodies) result in similar nuclear morphology phenotypes, disrupting their typical morphology and distribution pattern [71]. These findings suggest a dependence of Me31B-containing P-bodies on other RNP structures like U bodies in RNP assembly and hint at potential collaboration in the intracellular trafficking of snRNPs and mRNPs [71].

## Me31B in the male germline

Research on Me31B's role in the male germline is limited compared to that in females. However, recent studies have shown that this maternal gene also functions in the male germline cells. In one study, Me31B was found to be expressed in male testis cells like germline stem cells (GSCs), spermatogonia (SG), and spermatocytes [72]. Me31B was observed in cytoplasmic P-bodies, where it co-localizes with Decapping protein 1 (Dcp1) and exoribonuclease Pacman (PCM), both of which are involved in RNA degradation pathways. The study suggested that Me31B and Dcp1 work together to bind mRNAs and present them for degradation by PCM, which is required for male *Drosophila* spermatogenesis and fertility. Interestingly, the co-localization pattern of Me31B-Dcp1-PCM in male cells was similar to that seen in P-bodies in female nurse cells [32,73], indicating a potentially common mRNA regulation pathway mediated by Me31B-containing P-bodies in both sexes.

In a second study, Me31B’s role in maintaining *Drosophila* male GSC homeostasis was revealed [74]. The study indicated that Me31B is vital in preventing excessive de-differentiation of spermatogonia back into GSC, which may result from the failure in downregulating the expression of the GSC-maintenance and germ cell identity regulator *nos*. The study proposed a model explaining Me31B's role in GSC homeostasis. The model suggests that *nos* mRNA is initially translated in GSCs and their immediate daughter cells, which helps maintain the GSC’s stem cell status. Then, as GSCs give rise to SGs, Me31B binds and translationally represses *nos* mRNA, causing a Nos protein level decrease in the SGs, thereby promoting SG differentiation. This mechanism of action bears resemblance to the function of Me31B in suppressing *nos* mRNA translation during early embryo development, hinting at a comparable role of Me31B in regulating germline mRNAs in both sexes.

According to a third study, Me31B is associated with eIF4E-3, a specific isoform of Eukaryotic translation initiation factor 4E that is expressed in *Drosophila* testis and essential for spermatogenesis [75]. The research demonstrated that the two proteins co-express, co-immunoprecipitate, and co-localize in male germline cells such as spermatocytes. The Me31B–eIF4E-3 interaction connects the repressive function of Me31B to the regulation of translation.

## Me31B in the soma

Beyond the germline, it is noteworthy that Me31B also plays pivotal biological roles in somatic cells, with a particular emphasis on its involvement in neuronal functions. In fact, both *Drosophila* and mammalian neurons contain dynamic, cytoplasmic RNP granules known as ‘neuronal granules’ [76–80] that share many similarities with germline RNPs in terms of morphology, composition, and function. These granules often contain RNA regulators such as Me31B, FMRP (dFMR1 in *Drosophila*), Staufen (Stau), and ATX-2 [32,81], and appear to be involved in the storage and regulation of translationally repressed mRNAs [82–87]. In the following paragraphs, we will examine a range of studies (conducted both in cell cultures and brains) that explore Me31B’s impact on neuron physiology, development, and neuronal mRNA metabolism.

In primary culture *Drosophila* neurons, the overexpression of Me31B leads to abnormal down-regulation of high-order dendritic complexity. Conversely, the knockdown of Me31B yielded opposite effects, with all phenotypes dependent on Me31B's DEAD-box motif. These results suggest Me31B’s important role in dendrite morphogenesis [32].

In adult *Drosophila* brains, Me31B is present in several types of neuronal cells, including olfactory projection neurons, local interneurons, pacemaker neurons, mushroom-body neurons, and glial cells [88]. In olfactory projection neurons (PNs), Me31B is essential for the translational repression of CaMKII reporter mRNA, which is a miRNA-regulated, synapse-localizing mRNA. Additionally, the *me31B* gene and its genetic interaction with *atx2* and *dFMR1* are necessary for long-term olfactory habituation (LTH), a behavior trait involving olfactory PNs, and LTH-associated neuron structural plasticity [89–91]. In circadian pacemaker neurons, Me31B associates with and genetically interacts with *atx2* to maintain rhythmic circadian behaviors. This process is hypothetically linked to the repression functions of an Atx2-Me31B-NOT1 complex [92]. In mushroom body γ neurons (neurons involved in learning and memory), Me31B-containing neuronal granules condense, recruit repressor protein Imp and its target mRNA *profilin*, and inhibit *profilin* translation during neuron aging [93]. Conversely, the granules de-condense, release Imp and *profilin* mRNA, and de-repress *profilin* translation upon neuron stimulation by a biogenic peptide [94].

The Me31B ortholog, DDX6, can be located in the hippocampal neurons within mammals. As the neurons mature, DDX6-containing neuronal granules will disassemble into smaller-sized granules, but they will re-assemble into large granules if synaptic activities are inhibited. Additionally, DDX6 plays an important role in synaptic functions such as the appropriate clustering of a postsynaptic marker protein in neuronal dendrites and the frequency of Ca^2+^ peaking [95].

Me31B is not only found in neurons but also non-neuron soma. Me31B plays a crucial role in the cytoplasmic foci formation of core P-body components Dcp1 and Pcm in *Drosophila* wing imaginal discs (undifferentiated precursor cells of *Drosophila* wing). It also helps in the recruitment of repressor dFMR1 to P-bodies, and *bantam* and *miR2* miRNA- mediated translational repression [81]. In *Drosophila* eyes, Me31B, along with its partner repressor Tral, is needed for a *dFMR1* gene-mediated ‘rough eye’ phenotype [32].

Numerous studies examined in this section have provided convincing evidence that Me31B, or its orthologs like DDX6, plays a critical role in regulating neuron physiology, development, and synaptic activities. Me31B likely accomplishes this by forming and modifying neuronal RNP granules, which in turn regulates of the translation of neuronal mRNAs in a spatiotemporal manner. Storage, localization, repression, activation, and decay of these transcripts enable the neurons to respond appropriately to intracellular and extracellular signals [95–97]. This mode of action of Me31B in neuronal (and other somatic) granules is similar to what was observed in the germline, indicating that Me31B may be a necessary common component for the repressing RNPs in various cell types and species.

## How does Me31B affect target mRNAs?

**Figure 4:**
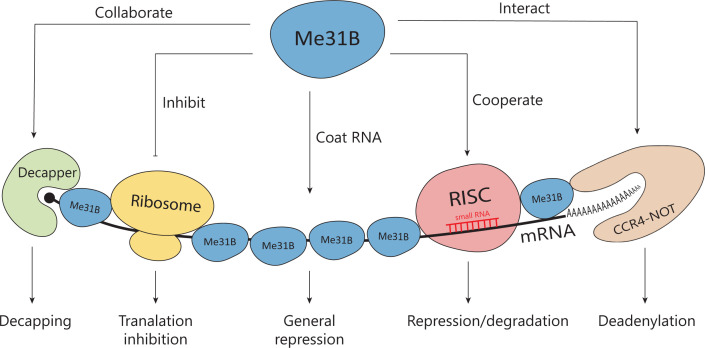
A schematic illustration of the mechanisms of how Me31B affects target mRNAs In this model, Me31B shows various ways of influencing its target mRNA. First, it oligomerizes and coats the mRNA, functioning as a general repressor. Second, it cooperates with RISC, a small RNA-equipped mRNA silencing machinery, in mRNA degradation/repression. Third, Me31B collaborates with decapping factors (Decapper) to facilitate mRNA decapping. Additionally, it physically interacts with CCR4-NOT complex, which induces mRNA deadenylation. Finally, Me31B may directly interact with ribosomes, impeding mRNA translation. Notably, these interconnected mechanisms of action may operate independently or synergistically in a unified RNA regulation pathway.

Many studies have identified Me31B as a general repressor in various RNPs, yet the exact means by which the protein affects target mRNA remains elusive. A growing body of evidence suggests Me31B promotes RNA storage, repression, and degradation through a network of interrelated mechanisms, including direct RNA coating, interaction with RNA-induced silencing complex (RISC), decapping factors, deadenylation complexes, and ribonucleases, as well as interfering with ribosomes (a schematic illustration showed in Figure 4). Given the limited research on *Drosophila* Me31B, we also draw upon studies of its orthologs (such as DDX6/RCK in humans, p54 in mice, Xp54 in frogs, CGH-1 in worms, Dhh1p in yeast) to shed light how this conserved family of helicases affects target mRNAs.

### RNA coating

According to the following studies, it is believed that Me31B functions as a repressor of target mRNAs by forming oligomers on them, coating them, and potentially shielding them from translation machinery. One study proposed that Me31B-Tral repressor dimers polymerize along *nos* mRNA [54]. Another study utilizing an Xp54 RNA-tethering assay found that Xp54 oligomerizes on mRNA in an RNA- and helicase activity-dependent manner [98]. Similarly, in a binding assay with recombinant DDX6 proteins, DDX6 polymerized and bound to single-stranded RNA targets [99].

### Participation in RISC-mediated pathways

RISCs are cellular complexes that play a vital role in the mRNA regulation pathway. Equipped with small RNAs, these complexes guide themselves to complementary or partially complementary target mRNAs, using effector proteins like Argonaute (AGO) to achieve translational repression or degradation [100–103]. RISC-mediated RNA regulation pathways rely on Me31B, or its equivalents in other organisms [104–111], to facilitate relevant mRNA degradation events such as P-body assembly [30,105,109,112–114], mRNA decapping [41,115–118], deadenylation [38,116,119–121], and nuclease digestion [47,88,107,122,123]. While human DDX6 and fly Me31B act as repressors for RISC-targeted mRNAs, they do not cleave the mRNAs (which is usually done by nucleases like AGO) [104]. This is consistent with Me31B’s role as a general repressor, rather than a degrader. When working with decapping factors, yeast Dhh1p promotes efficient mRNA decapping and directly interacts with main decapping enzymes Dcp1/Dcp2 and other decapping activators like Pat1 and Edc3 [115,116]. However, Dhh1p is believed to work by repressing translation initiation, rather than stimulating the decapping enzymes (Dcp1/Dcp2) [41]. During mRNA deadenylation, Me31B interacts physically with a highly conserved deadenylation complex CCR4-NOT in yeast, fruit fly, and human cells [38,116,119–121]. It is important to mention that in humans, DDX6 binds to the NOT1 protein which acts as the assembly scaffold of the CCR4-NOT complex. This binding stimulates DDX6's ATP hydrolysis activity [124] and is crucial for RNA repression and silencing [38,110,124]. This suggests that DDX6’s ATPase activity plays a significant role in its function as a repressor.

### Interaction with ribosomes

Research has shown that yeast Dhh1p interacts with ribosomes, potentially modulating translation. Recombinant Dhh1p at high concentrations can prevent the formation of translation preinitiation complexes [41]. Dhh1p also binds preferentially to mRNAs containing non-optimal codons (i.e., low translation efficiency mRNAs), leading to an accumulation of slow-moving ribosomes and the mRNA’s degradation [125,126]. Additionally, Dhh1p can physically interact with eukaryotic ribosomes and certain ribosomal RNAs [125].

It should be noted that Me31B’s mechanisms of action discussed in this section (Figure 4), such as RNA coating, interacting with RISC, decapping enzymes, deadenylation complexes, degradation enzymes, and modulating ribosomes, are interconnected and often converge as sequential processing steps involved in RNA regulation.

## Perspectives and unanswered questions

There are still a lot of questions when it comes to the molecular biology of the Me31B. We will delve into several of them, as we believe their answers will give us a better understanding of this conserved helicase family (Box 1).

Box 1 **Unanswered questions**

1. What is the role of Me31B in an RNP?
  - If Me31B is a repressor, scaffold, and/or remodeler, how does Me31B transit between different functions to render different fates to mRNAs.
2. Does Me31B’s function rely on the protein’s localization in RNPs?
3. What’s Me31B’s molecular level working mechanism?
  - How does Me31B interact with RNAs and protein partners?
  - What are the biological implications of these interactions?
  - How does Me31B adapt to changing cellular contexts to remodel the RNPs and output the appropriate functions?

What is the role of Me31B in an RNP? Based on the research reviewed, Me31B has been identified as a versatile player, serving as a general repressor, an RNP scaffold/hub, and an RNP remodeler. As a general repressor, Me31B exhibits low RNA-binding specificity and inhibits translation without modifying the RNA. As an RNP scaffold/hub, Me31B coordinates the assembly of a repressor RNP on a target RNA by recruiting and/or being recruited by other factors. Me31B’s interaction with these factors will determine the RNA’s fate. As an RNP remodeler, Me31B can dynamically alter an RNP’s composition, morphology, and function in response to developmental changes and cellular contexts. This allows Me31B-bound mRNAs to experience different outcomes, including degradation, repression for storage, or release for translational activation. This naturally raises the question of how Me31B can transit between these different functions. An earlier study proposed an intriguing model suggesting that Me31B alters RNP functions by component exchanges [88]. This idea is supported by previous research indicating that Me31B’s C-terminal RecA-like domain binds exclusively to different partners, including Edc3, Tral, ATX2, and NOT1 [33,92,127]. The binding of Edc3 with DDX6 leads to the assembly of a decapping complex (containing decapping factors Dcp1, Edc4, and Dcp2), while the binding of Tral with DDX6 results in a repressing complex (containing eIF4E binding protein Cup, which can inhibit the decapping of bound mRNA). Such a transition in the fate of bound mRNA would be significant [128]. Furthermore, Pat and Edc3's binding interferes and competes with the RNA binding of yeast Dhh1p [127], suggesting a flexible Dhh1p–RNA interaction and a possible mechanism for RNA release.

The role of Me31B and its dependence on the protein’s localization into RNP granules has sparked much debate. However, a recent study shed light on this topic by introducing a *Drosophila* strain carrying mutations that prevent Me31B’s localization to RNP granules [19]. By disrupting Me31B’s FDF-binding motif, the mutant Me31B is dispersed throughout the germline instead of aggregating onto RNP granules. Interestingly, this dispersed Me31B only slightly decreased fertility and did not appear to affect *Drosophila* oogenesis, embryogenesis, or the formation of germline RNPs (as indicated by other maker proteins Tral and Cup). These findings suggest that Me31B’s localization to RNP granules may not be necessary for its function. One possible explanation is that Me31B is abundant enough in the germline to interact with the RNPs and provide essential mRNA regulation functions. It would be valuable to investigate whether this concept applies to other species or cell systems.

Understanding the full working mechanism of Me31B is a complex yet crucial endeavor. How does Me31B interact with RNAs and various protein partners, and what are the implications of the interactions? Furthermore, how does Me31B adapt to changing cellular contexts to remodel the RNPs and output the appropriate functions? Although past research has provided some answers, a comprehensive understanding of Me31B’s working mechanism remains elusive. One promising future approach may involve the systematic generation of weak, target-motif-mutation alleles of *me31B*. These tools could prove invaluable in uncovering the individual roles of different Me31B motifs, ultimately leading to a more complete understanding of the protein’s overall mechanism and function.

## Data Availability

No research data was generated in the article.

## Abbreviations

AGO: Argonaute
Dcp1: Decapping protein 1
GSC: germline stem cell
IDR: intrinsically disordered region
Me31B: Maternally Expressed at 31B
PCM: Pacman
RNP: ribonucleoprotein
SG: spermatogonia
SRE: Smaug Recognition Element
